# Preliminary Study on the Efficient Electrohysterogram Segments for Recognizing Uterine Contractions with Convolutional Neural Networks

**DOI:** 10.1155/2019/3168541

**Published:** 2019-10-13

**Authors:** Jin Peng, Dongmei Hao, Haipeng Liu, Juntao Liu, Xiya Zhou, Dingchang Zheng

**Affiliations:** ^1^College of Life Science and Bioengineering, Beijing University of Technology, Intelligent Physiological Measurement and Clinical Translation, Beijing International Platform for Scientific and Technological Cooperation, Beijing 100024, China; ^2^Medical Technology Research Centre, Faculty of Health, Education, Medicine and Social Care, Anglia Ruskin University, Chelmsford CM1 1SQ, UK; ^3^Department of Obstetrics, Peking Union Medical College Hospital, Beijing 100730, China

## Abstract

**Background:**

Uterine contraction (UC) is the tightening and shortening of the uterine muscles which can indicate the progress of pregnancy towards delivery. Electrohysterogram (EHG), which reflects uterine electrical activities, has recently been studied for UC monitoring. In this paper, we aimed to evaluate different EHG segments for recognizing UCs using the convolutional neural network (CNN).

**Materials and Methods:**

In the open-access Icelandic 16-electrode EHG database (122 recordings from 45 pregnant women), 7136 UC and 7136 non-UC EHG segments with the duration of 60 s were manually extracted from 107 recordings of 40 pregnant women to develop a CNN model. A fivefold cross-validation was applied to evaluate the CNN based on sensitivity (SE), specificity (SP), and accuracy (ACC). Then, 1056 UC and 1056 non-UC EHG segments were extracted from the other 15 recordings of 5 pregnant women. Furthermore, the developed CNN model was applied to identify UCs using different EHG segments with the durations of 10 s, 20 s, and 30 s.

**Results:**

The CNN achieved the average SE, SP, and ACC of 0.82, 0.93, and 0.88 for a 60 s EHG segment. The EHG segments of 10 s, 20 s, and 30 s around the TOCO peak achieved higher SE and ACC than the other segments with the same duration. The values of SE from 20 s EHG segments around the TOCO peak were higher than those from 10 s to 30 s EHG segments on the same side of the TOCO peak.

**Conclusion:**

The proposed method could be used to determine the efficient EHG segments for recognizing UC with the CNN.

## 1. Introduction

Uterine contraction (UC) is the tightening and shortening of the uterine muscles. UC can reflect the progress of pregnancy towards delivery and is a major observation for estimating the approach of delivery [1]. Electrohysterogram (EHG), which reflects uterine electrical activities, is a promising noninvasive technology for external UC monitoring [2]. However, it is still ambiguous which EHG segments are appropriate for recognizing UC.

Currently, four methods have been proposed to assess UCs. Manual palpation, which identifies UC by palpating the parturient abdomen over the uterine corpus, requires the constant bedside presence of a trained operator [1]. Internal uterine catheter (IUPC) is limited by its invasiveness and the need for ruptured membranes [3]. External tocodynamometry (TOCO) is noninvasive, but its recording quality depends on correct position of the sensor on the maternal abdomen and is influenced by maternal movements and the amount of subcutaneous fat [4]. Recently, EHG measurement has been considered a noninvasive method and an alternative approach of TOCO to monitor UC [5].

EHG features have been investigated to distinguish between UCs and non-UCs (non-uterine contractions) [6, 7]. These features have been extracted by power spectral density, wavelet packet decomposition [8], autoregression model [9], and other signal processing methods in the time and the frequency domain [1, 7]. Nonlinear processes have also been involved in generating UCs because of the complex interactions between billions of myometrium cells [10]. Therefore, nonlinear methods including time reversibility, sample entropy, Lyapunov exponents and delay vector variance [11], nonlinear interdependencies [10], and multifractal analysis [12, 13] are useful for EHG analysis. Some advanced algorithms including the Hilbert transform, cross-correlation [14], correlation coefficient *H*^2^ [5], mutual correlation dimension, cross-approximate entropy [15], and dynamic cumulative sum [16] have also been proposed for UC detection. Besides, classifiers including the support vector machine [17], random forest, and artificial neural network [7] have been developed for automatic UC detection using TOCO, cardiotocogram [18], and EHG signals. Even though some convincing results have been reported, there were discrepancies between them because of different data sources, feature selection algorithms, and classifiers applied [19, 20].

The convolutional neural network (CNN) has recently been applied to obstetrics and gynecology [21] for classification of the fetal heart rate [22], electromyography [23], and electrocardiogram signals [24]. The CNN is a type of machine learning which can classify images and time series without additional feature extraction and selection and produce state-of-the-art recognition results. The outstanding classification capability of the CNN provides possibilities for detecting UCs with EHG images.

The purpose of this study is to investigate the EHG segments appropriate for identifying UC. A CNN will be developed by EHG segments of 60 s and then utilized to evaluate EHG segments of 10 s, 20 s, and 30 s relative to the TOCO peak.

## 2. Materials and Methods

**Figure 1:**
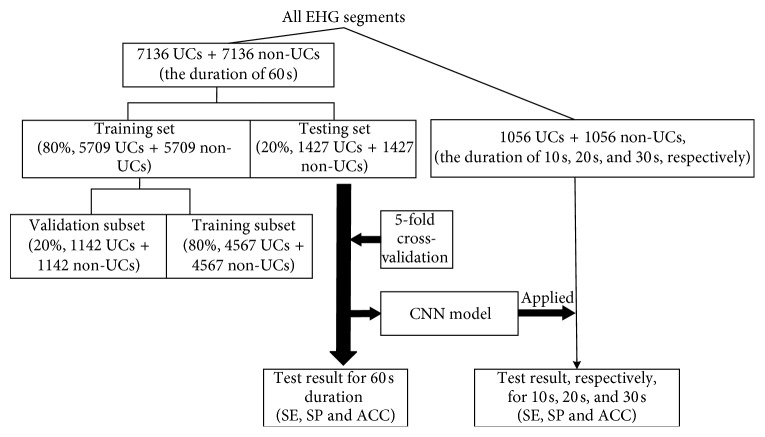
Flow chart of our proposed method. UC = uterine contraction; non-UC = non-uterine contraction; SE = sensitivity; SP = specificity; ACC = accuracy.

EHG signals were first manually segmented into UCs and non-UCs based on UC annotations and TOCO signals. 7136 UCs and 7136 non-UCs of 60 s duration were extracted from 107 recordings of 40 pregnant women and used to establish a CNN model. Then, 1056 UCs and 1056 non-UCs were extracted from the other 15 recordings of 5 pregnant women. In particular, the EHG segments of 10 s, 20 s, and 30 s were classified as UC and non-UC using the established CNN model. The EHG segments of different durations were evaluated based on their sensitivity (SE), specificity (SP), and accuracy (ACC). In this study, a UC was divided into several small segments, and those with higher SE and ACC were considered efficient EHG segments for recognizing UC. The details of each step are shown in Figure 1.

### 2.1. Icelandic 16-Electrode EHG Database

**Figure 2:**
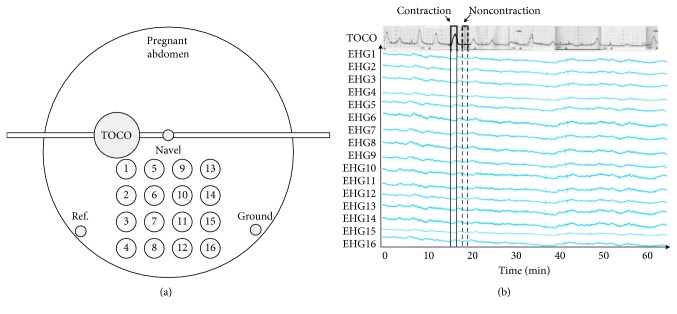
(a) The ideal configuration of EHG electrodes (the reference and ground electrodes were not standardized to certain sides for the recordings). (b) An example of 16-channel EHG and TOCO signals from the Icelandic 16-electrode EHG database. Ref.: reference electrode.

The open-access Icelandic 16-electrode EHG database contained 122 EHG recordings performed on 45 pregnant women, and some of them were measured more than once at Akureyri Primary Health Care Centre and Landspitali University Hospital between 2008 and 2010 in Iceland [25]. The database also provided simultaneously recorded tocographs, annotations of events, and obstetric information of participants. The participants had normal singleton pregnancies without any known preterm birth risk factors. A grid of 4 × 4 electrode was placed on the abdomen with the reference and ground electrodes on each side of the body (not standardized), as shown in Figure 2(a). Recordings were performed in the third trimester (112 recordings) and during labor (10 recordings). The average recording durations for pregnancy and labor were 61 and 36 min. The EHG signals were sampled at 200 Hz.

### 2.2. EHG Signal Preprocessing and Segmentation

EHG signals were downsampled at 20 Hz and preprocessed by a 5th order Butterworth bandpass digital filter (0.1∼4 Hz) to remove the unwanted interference [20, 26].

Each EHG signal was manually divided into UC and non-UC segments based on the UC annotation and TOCO signal [5, 27]. The duration of the UC segment was symmetric around the TOCO peak for easy identification [4, 28]. The corresponding non-UC was extracted between two UCs, as shown in Figure 2(b). In total, 7136 UCs and 7136 non-UCs of 60 s duration were extracted and confirmed by two clinicians. The extracted segments were discarded in case any clinician disagreed.

**Figure 3:**
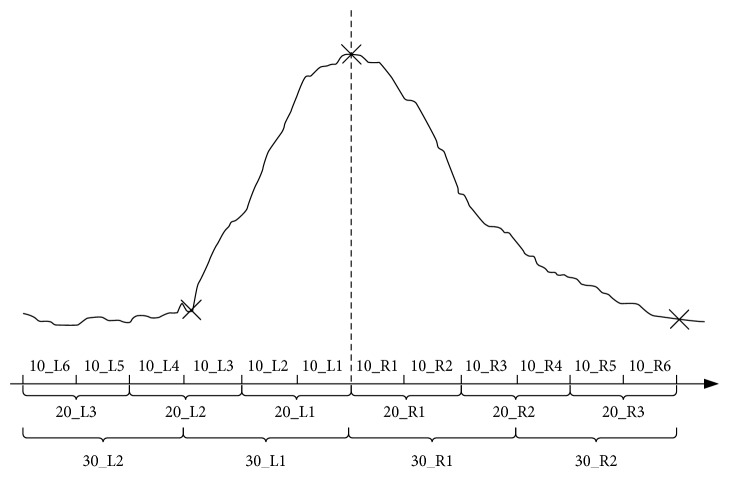
An example of the EHG segment with a different duration and position corresponding to the TOCO signal. The start, peak, and end points of the TOCO signal are marked by cross (“×”). The corresponding EHG segment is divided into different durations of 10 s, 20 s, and 30 s and named 10_L1∼6 and 10_R1∼6, 20_L1∼3 and 20_R1∼3, and 30_L1∼2 and 30_R1∼2, respectively.

Then, the EHG segments with the duration of 10 s, 20 s, and 30 s were extracted from the left and right sides of the TOCO peak, as shown in Figure 3. Considering the time difference between the EHG recordings, annotations, and tocographs [28, 29], twelve 10-second EHG segments (10_L1∼6 and 10_R1∼6), six 20-second EHG segments (20_L1∼3 and 20_R1∼3), and four 30-second EHG segments (30_L1∼2 and 30_R1∼2) with a total 120 s duration were extracted to contain UC segments as many as possible. 1056 UCs and 1056 non-UCs with different durations were extracted.

Finally, all EHG segments were saved as images and normalized to 482 × 482 pixels by resizing. Sixteen EHG images were obtained from 16-channel recordings for each UC and non-UC.

### 2.3. Convolutional Neural Network for Classification of EHG Segments

**Figure 4:**
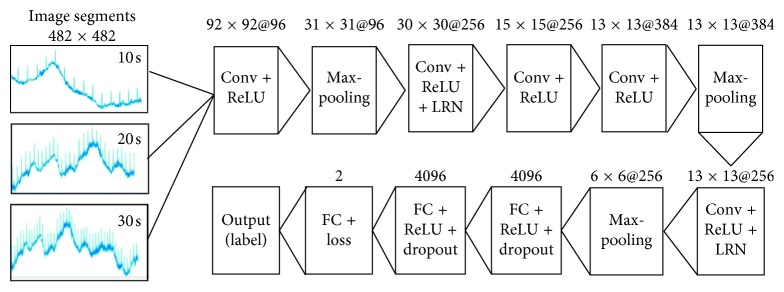
CNN architecture with 11 layers including the Conv, max-pooling, and FC layers.

The CNN is a specialized deep neural network for processing 1D time series and 2D images [24]. In this study, the CNN consisted of convolutional (Conv), max-pooling, fully connected (FC), local response normalization (LRN), dropout, and softmax layers and a rectified liner unit (ReLU), as shown in Figure 4. The Conv layer with the image size of length *l* and width *w* and the number of filters (*m*) denoted by *l* × *w*@m was used to extract features of the input image. The max-pooling layer downsampled the feature map and reduced the computational complexity. The number of neurons in the FC layer was denoted by num_output.

Every Conv and every FC were followed by a ReLU [24, 30] which could be activated to speed up the training process. Behind a ReLU, the LRN layer detected high-frequency features and assigned them with large weights [31]. The parameters in the LRN layer were set as follows: the local_size value of 5, *α* value of 0.0001, and *β* value of 0.75 [32]. The dropout layer with half connection could reduce overfitting and improve regularization [30]. The batch gradient descent algorithm was applied to facilitate the CNN converge with the global optimum. Finally, the FC layer was connected to the softmax function (loss, shown in Table 1) to obtain the last output [22].

Stride refers to the number of samples that the filter slides over the input image. In the first layer, the size of the input image changed from 482 × 482@96 to 92 × 92@96 when the kernel was set to 27 and stride was set to 5. Then, the image size decreased from 92 × 92@96 to 31 × 31@96 after the max-pooling layer. Subsequently, the size of the image was processed with a stride of 1, kernel of 2, and max-pooling layer to reduce its size from 30 × 30@256 to 15 × 15@256. After the third Conv layer, the image size was further reduced to 13 × 13@384. The Conv and max-pooling layers were once again performed on the output neuron of 13 × 13@256 and 6 × 6@256, respectively. These were followed by FC layers with 4096 neurons. The final FC layer consisted of two neurons to classify UC and non-UC. The detailed parameters, including the kernel, stride, weight, and bias initialization of the CNN, are listed in Table 1, based on prior knowledge and manual tuning to achieve a satisfactory training result.

Different results were produced because of different hyperparameter values at each training of the CNN [22]. The repetition of each experiment process is called an “iteration” [33]. The standard deviation was set to 0.1, and some small positive values (0, 1) were added to the bias to avoid dead nodes [22]. The dropout (dropout_ratio) was set to 0.5 to gain the best results. Based on the results from the preliminary test, fine tuning was performed with the learning rate of 0.001, weight decay of 0.0005, learning rate drop factor of 0.1, learning rate drop period of 10, momentum of 0.9, gamma of 0.1, and maximum iteration of 20000. The hyperparameters for training the CNN are shown in Table 2.

The CNN was run on a workstation with Linux Ubuntu 18.04 LTS Operating System and NVIDIA 1080 Ti GPU. The development environment was the CAFFE net framework, and the development language was MATLAB and Python.

### 2.4. Evaluation of CNN Model

A fivefold cross-validation was utilized to evaluate the performance of the established CNN [34]. 7136 UCs and 7136 non-UCs were equally divided into five subsets, four of which were used to train the CNN model and the other was used to test the CNN model. This process was repeated five times. Furthermore, the training set included training (80% of the training set) and validation (20% of the training set) subsets, in which the validation subset was used to tune the hyperparameters of the CNN.

SE, SP, and ACC were used to evaluate the classification performance, which were calculated as follows:

(1) $$\begin{array}{c} \text{SE}=\frac{\text{TP}}{\text{TP}+\text{FN}},\\ \text{SP}=\frac{\text{TN}}{\text{FP}+\text{TN}},\\ \text{ACC}=\frac{\text{TP}+\text{TN}}{\text{FP}+\text{TN}+\text{TP}+\text{FN}}, \end{array}$$

where TP (true positive) and TN (true negative) are the numbers of UC and non-UC EHG segments that were correctly classified and FP (false positive) and FN (false negative) are the numbers of UC and non-UC EHG segments that were falsely classified. The results of SE, SP, and ACC from the fivefold cross-validation were calculated and averaged to evaluate the CNN.

Furthermore, the established CNN was utilized to classify EHG segments of 10 s, 20 s, and 30 s to distinguish between UCs and non-UCs. The results of SE, SP, and ACC could indicate which EHG segments were efficient for recognizing UC with the CNN.

### 2.5. Statistical Analysis

One-way ANOVA with Tukey's method was performed using the software SPSS 22 (SPSS Inc.) to compare SE, SP, and ACC between EHG segments with the same duration. A *p* value less than 0.05 was considered statistically significant.

## 3. Results

### 3.1. Evaluation of CNN Performance with EHG Segments of 60 s Duration

With the training set, the ACCs of five validations were 0.99, 1.00, 0.99, 0.98, and 0.99 and the loss ratios were 0.07%, 0.01%, 0.11%, 0.09%, and 0.08%. With the testing set, the averaged SE of 0.82, SP of 0.93, and ACC of 0.88 were achieved, as shown in Table 2.

### 3.2. Evaluation of CNN Performance Using EHG Segments of Different Durations

Table 3 shows the results from twelve 10-second EHG segments (10_L1∼6 and 10_R1∼6), six 20-second EHG segments (20_L1∼3 and 20_R1∼3), and four 30-second EHG segments (30_L1∼2 and 30_R1∼2). As indicated in Table 3, the values of SE and ACC from the EHG segments around the TOCO peak (10_L1 and 10_R1, 20_L1 and 20_R1, and 30_L1 and 30_R1) were higher than those from the other segments of the same duration (10_L2∼6 and 10_R2∼6, 20_L2∼3 and 20_R2∼3, and 30_L2 and 30_R2) (comparisons on both sides of the TOCO peak, respectively). In contrast, the values of SP were similar among EHG segments of different durations. Besides, the values of SE from 20 s EHG segments around the TOCO peak (20_L1 and 20_R1) were higher than those from 10 s to 30 s EHG segments on the same side of the TOCO peak (10_L1, 30_L1 and 10_R1, 30_R1).

### 3.3. Comparison of SE between Different EHG Segments

**Figure 5:**
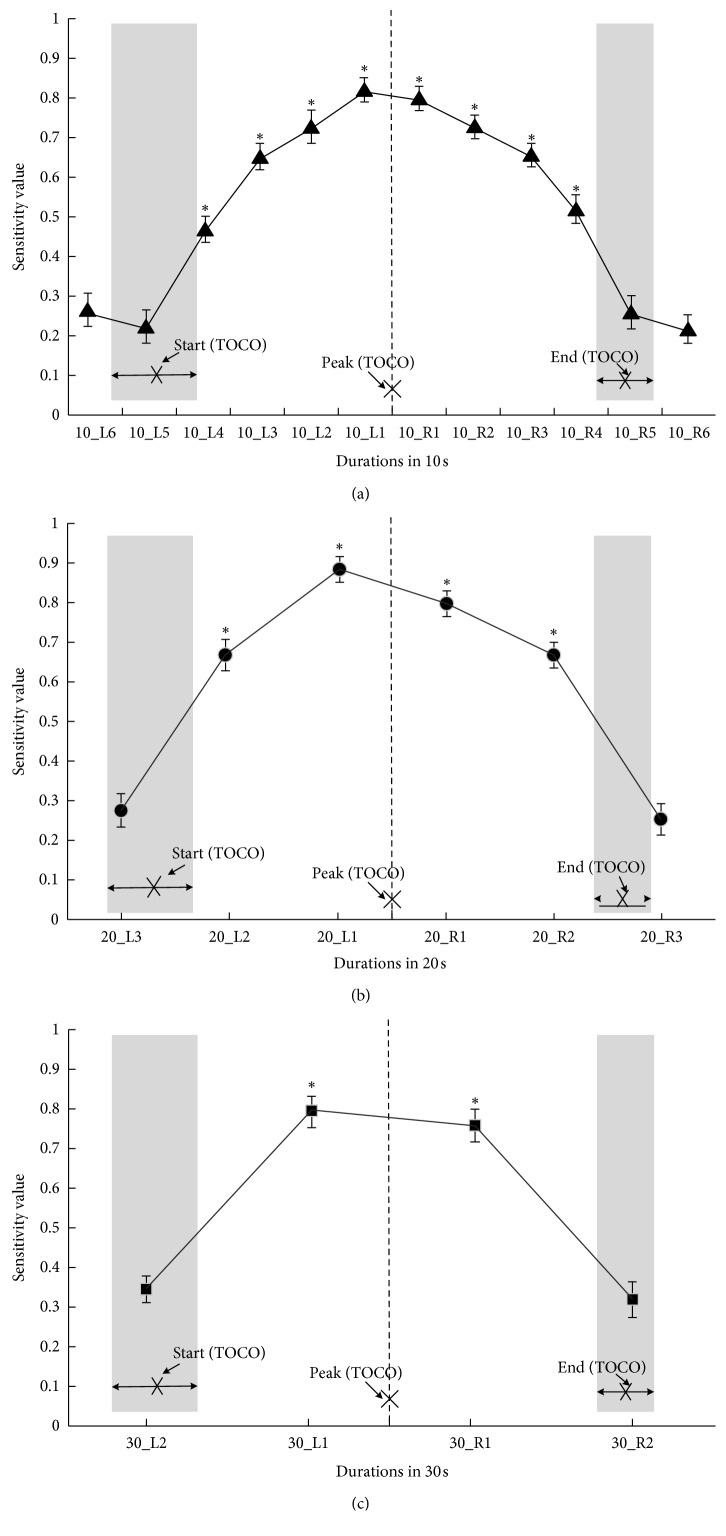
SE of EHG segments with different durations. (a) SE from 10 s EHG segments. (b) SE from 20 s EHG segments. (c) SE from 30 s EHG segments. ^*∗*^*p* < 0.05: significant difference compared with 10_L6, 20_L3, and 30_L2, respectively, in (a), (b), and (c). The range of the time difference between TOCO and EHG segments is highlighted by grey shades. The cross (“×”) represents the mean of the time difference.

Figure 5 shows SE from EHG segments of different durations. The range of the time difference between TOCO and EHG segments of UC at the start and end points is highlighted by shades of grey, and the mean of the time difference is denoted by a cross. In terms of 10 s duration, the SE values from 10_L1∼4 and 10_R1∼4 were significantly larger than those from 10_L6 (*p* < 0.05). In terms of 20 s duration, the SE values from 20_L1∼2 and 20_R1∼2 were significantly larger than those from 20_L3 (*p* < 0.05). In terms of 30 s duration, the SE values from 30_L1 and 30_R1 were significantly larger than those from 30_L2 (*p* < 0.05). No significant difference was found between the start and end points (*p* > 0.05).

## 4. Discussion

In this paper, a CNN model was built with UC and non-UC EHG segments of 60 s duration and then applied to recognize UCs using different EHG segments (different durations and different positions relative to a TOCO peak). The results indicate the efficient EHG segments that could be used to recognize UCs and monitor pregnancy progress in the future. To the best of our knowledge, this is the first study on the duration and position of EHG segments in distinguishing between UCs and non-UCs.

The EHG segment corresponding to UC was investigated with the TOCO peak as a reference, which was easier to identify than the start and end points. In this study, the EHG segments from different channels and different gestational weeks were mixed together because of the small dataset. The duration of the EHG segment was selected based on the following consideration: (i) the duration of 10 s performed the best in identifying and tracking uterine activity across subjects [14] and (ii) most of the UC durations were no more than 60 s based on the Icelandic EHG database and clinical experience.

Several EHG analysis methods including the nonlinear correlation coefficient *H*^2^ [5], cross-correlation [14], and root-mean-square envelope [28] have been proposed to improve the accuracy of UC detection. However, none of them concerned about the effects of the duration and position of the EHG segment on UC detection. Table 3 and Figure 5 show that EHG segments around the TOCO peak (10_L1 and 10_R1, 20_L1 and 20_R1, and 30_L1 and 30_R1) achieved higher SE and ACC than the others, indicating that they are more efficient for recognizing UCs. Furthermore, the SE of 20 s EHG segments (20_L1 and 20_R1) was better than that of other duration segments. The duration of 10 s and 30 s was supposed to contain a part of UC or additional non-UC which may influence its identification ability. We also observed different results on both sides of the TOCO peak, which might be due to the UC variation during pregnancy [35] and the imprecise synchronization between TOCO and EHG.

In this study, we focus on comparing EHG segments on each side of the TOCO peak separately because the rising and descending phases of the EHG segment reflect the tension and relaxation of the myometrium and may have different effects on recognizing UCs. Furthermore, recognition of UCs using EHG segments covering both sides of the TOCO peak (similar to 10_L1 + 10_L2 + 10_R1 + 10_R2) is also indispensable, which has been investigated in our previous study [36].

At the current stage, the EHG segments corresponding to UCs and non-UCs were first manually extracted to train a CNN model. The established CNN could then be applied to EHG segments determined by our study to automatically recognize UC; thereafter, the manual segmentation is no longer required. The ability to differentiate UCs from non-UCs could be improved with the efficient EHG segments. The clinicians agreed that our proposed method is very promising and could be applied to long-term UC monitoring in practice.

The results were obtained from the combination of 16-channel EHG signals because of the small dataset at present. We will work at reducing the number of EHG-recording electrodes for clinical application. The current CNN model was built by limited images from different gestational ages, and the ability of recognizing UC may vary depending on gestational age. More data on different gestational ages could be collected to build models in scale-up studies to eliminate the influence of different gestational ages and improve the usability of the CNN technique. The signal-to-noise ratio (SNR) of the EHG also affected UC recognition. Therefore, the effects of the EHG channel, gestational week, and SNR will be investigated to further improve UC detection.

## 5. Conclusion

The proposed method could be used to determine the efficient EHG segments for recognizing UC with the CNN. The results showed the EHG segments around the TOCO peak achieved higher SE and ACC than the others with the same duration, which indicated that they are efficient for UC detection.

## Figures and Tables

**Table 1:** Detailed parameters used for all the layers of the CNN model.

Layer	Type	Kernel size	Other layer parameters
1	Conv + ReLU	27	Strides = 5, num_output = 96
2	Max-pooling	2	Strides = 3
3	Conv + LRN + ReLU	2	Strides = 1, local_size = 5, *α* = 0.0001, *β* = 0.75, num_output = 256
4	Max-pooling	2	Strides = 2
5	Conv + LRN + ReLU	3	Strides = 1, num_output = 384, local_size = 5, *α* = 0.0001, *β* = 0.75
6	Conv + ReLU	3	Strides = 1, pad = 2, num_output = 384
7	Conv + ReLU	3	Strides = 1, pad = 2, num_output = 256
8	Max-pooling	3	Strides = 2
9	FC + ReLU + dropout		Dropout_ratio = 0.5, num_output = 4096
10	FC + ReLU + dropout		Dropout_ratio = 0.5, num_output = 4096
11	FC		num_output = 2, activation = softmax

Pad is the padding number with leading zeros.

**Table 2:** Test results with EHG segments of 60 s duration.

Duration of the EHG segment		Resulting values				Calculating parameters		
		FP	FN	TP	TN	SE	SP	ACC
60 s	Fold1	97	241	1194	1338	0.83	0.93	0.88
	Fold2	92	253	1174	1335	0.82	0.94	0.88
	Fold3	107	252	1175	1320	0.82	0.93	0.87
	Fold4	92	254	1159	1321	0.82	0.93	0.88
	Fold5	116	251	1183	1318	0.82	0.92	0.88
	Average	504	1251	5885	6632	0.82	0.93	0.88

**Table 3:** Test results with EHG segments of different durations.

Different durations	Resulting values				Parameters		
	FP	FN	TP	TN	SE	SP	ACC
10_L6	107	793	263	949	0.25	0.90	0.57
10_L5	115	777	279	941	0.26	0.89	0.58
10_L4	162	534	522	894	0.49	0.85	0.67
10_L3	287	368	688	769	0.65	0.73	0.69
10_L2	190	308	748	866	0.71	0.82	0.76
10_L1	115	183	873	941	0.83	0.89	0.86
10_R1	124	206	850	932	0.80	0.88	0.84
10_R2	135	259	797	921	0.75	0.87	0.81
10_R3	261	349	707	795	0.67	0.75	0.71
10_R4	151	517	539	905	0.51	0.86	0.68
10_R5	268	887	169	788	0.25	0.84	0.55
10_R6	182	844	212	874	0.20	0.83	0.51
20_L3	241	726	330	815	0.31	0.77	0.54
20_L2	160	344	712	896	0.67	0.85	0.76
20_L1	130	108	948	926	0.90	0.88	0.89
20_R1	176	205	851	880	0.81	0.83	0.82
20_R2	169	371	685	887	0.65	0.84	0.74
20_R3	198	784	272	858	0.26	0.81	0.54
30_L2	210	687	369	846	0.35	0.80	0.58
30_L1	135	216	840	921	0.80	0.87	0.83
30_R1	154	238	818	902	0.77	0.85	0.81
30_R2	198	750	306	858	0.29	0.81	0.55

## Data Availability

The database used in this study is available for access via the following link: https://physionet.org/pn6/ehgdb/.
